# Progress in C-C and C-Heteroatom Bonds Construction Using Alcohols as Acyl Precursors

**DOI:** 10.3390/molecules27248977

**Published:** 2022-12-16

**Authors:** Feng Zhao, Bin Tan, Qing Li, Qi Tan, Huawen Huang

**Affiliations:** 1 Hunan Provincial Key Laboratory for Synthetic Biology of Traditional Chinese Medicine, School of Pharmaceutical Sciences, Hunan University of Medicine, Huaihua 418000, China; 2 Key Laboratory for Green Organic Synthesis and Application of Hunan Province, Key Laboratory of Environmentally Friendly Chemistry and Application of Ministry of Education, College of Chemistry, Xiangtan University, Xiangtan 411105, China

**Keywords:** alcohols, acyl, acylation, oxidative coupling, dehydrogenative coupling, amide

## Abstract

Acyl moiety is a common structural unit in organic molecules, thus acylation methods have been widely explored to construct various functional compounds. While the traditional Friedel–Crafts acylation processes work to allow viable construction of arylketones under harsh acid conditions, recent progress on developing acylation methods focused on the new reactivity discovery by exploiting versatile and easily accessible acylating reagents. Of them, alcohols are cheap, have low toxicity, and are naturally abundant feedstocks; thus, they were recently used as ideal acyl precursors in molecule synthesis for ketones, esters, amides, etc. In this review, we display and discuss recent advances in employing alcohols as unusual acyl sources to form C-C and C-heteroatom bonds, with emphasis on the substrate scope, limitations, and mechanism.

## 1. Introduction

Acyl-containing organic compounds, including ketones, esters, amides, and so forth, are a huge library of widespread chemical feedstocks which play a vital role in countless fields such as pharmaceuticals, natural products, advanced materials, and fine chemicals [1,2,3,4]. Based on their importance, significant achievements have been developed by chemists over the past decades. The classical routes to incorporate acyl group into aromatic compounds mainly rely on the Friedel–Crafts acylation reactions [5,6,7,8]. However, these methods often suffer poor selectivity, limited substrate scope, the requirement of stoichiometric moisture-sensitive Lewis acid and corrosive acyl chloride or anhydride, as well as concurrent waste production. Therefore, the development of effective and general methodologies for the selective incorporation of acyl groups into target structures has become a prevalent topic in organic synthetic chemistry. In recent years, transition-metal-catalyzed acylation reactions have attracted intensive attention and provided an efficient and straightforward synthetic route to access various acylated molecules in both academic and industrial areas [9,10,11]. Concomitantly, a number of simple and mild acylating reagents, such as aldehydes, carboxylic acids, esters, amides, etc., have been well explored and smoothly applied in multiple acylation transformations. Despite high efficiency, the disadvantages of these reagents should also be discussed. For instance, aldehydes are usually expensive and reactive. They are often prone to decomposing and being distilled or recrystallized prior to use in catalytic reactions. Moreover, a range of aldehydes are not naturally abundant or commercially available.

On the other hand, it is well known that alcohols are cheap, stable, low-toxic, naturally abundant, and commercially available. Thus, they are commonly used in the cross-coupling reactions [12], especially as oxygen-centered nucleophiles to synthesize ethers [13,14]. However, they are traditionally less used to serve as carbon-centered coupling partners. Considering the importance of these plentiful and economic chemicals in organic synthesis, alcohols can potentially act as ideal acyl precursors compared with the above reagents. Generally, the metal species react with the alcohol to yield the key alkoxide intermediate, followed by β-hydride elimination and reductive elimination to afford the final products and complete the catalytic cycle. With these strategies, a variety of alcohol acylation reactions can be found to afford a sustainable dehydrogenation manner for the construction of versatile molecules. In this review, we will focus our attention on the recent developments in utilizing alcohols as uncommon acyl sources through generally radical or organometallic couplings to construct C-C and C-heteroatom bonds, such as C-O, C-S, and C-N bonds, delivering ketones, esters, amides, and beyond. Notably, the studies mentioned in this article have been listed since 2010. Different metal catalytic systems (Pd, Rh, Ru, Cu, Ni, Mn, etc.) and reaction types are displayed and the versatile structural units for each are discussed, which are not mainly presented in previous works [15,16].

## 2. C-C Bond Construction

Acylation is a class of crucial C-C bond forming reaction which is widely involved in organic chemistry. One of its major applications is the formation of ketones, which are fundamental scaffolds found in various pharmaceuticals, agrochemicals, and natural products [17,18,19,20]. Typically, the generation of these valuable compounds relies on the addition of Grignard reagents to aldehyde, which is the most used reaction to form C−C bonds in the organic and medicinal chemistry. However, aldehyde is usually generated by the oxidation of alcohol precursor. Moreover, an additional step of oxidation is needed if ketone is the target product [21,22].

**Scheme 1:**
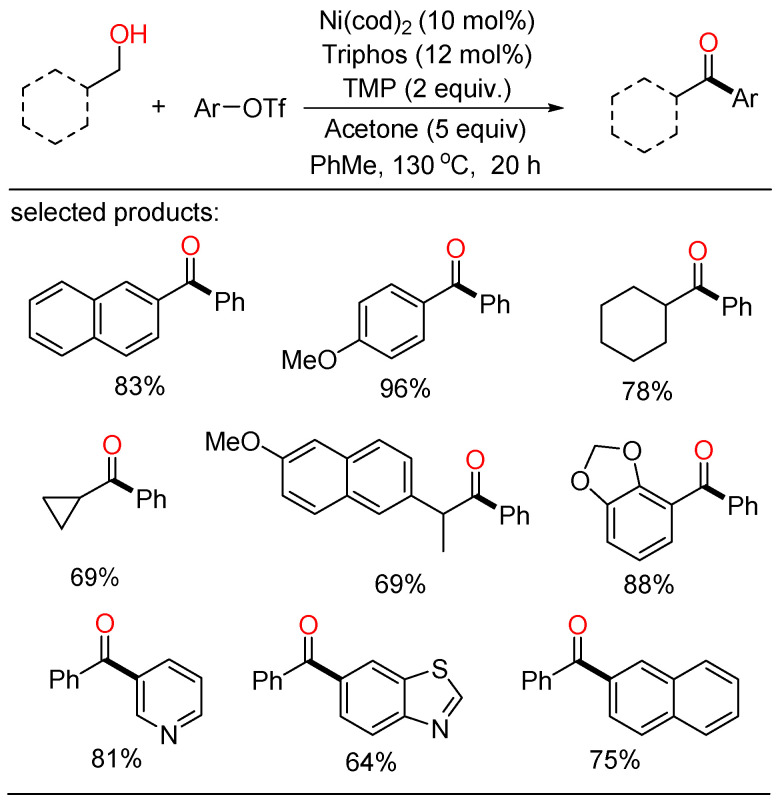
Ni-catalyzed cross-coupling of primary alcohols and organotriflates.

In 2019, Newman established a Ni-catalyzed cross-coupling of primary alcohols and organotriflates (Scheme 1) [23]. In this novel procedure, a variety of ketone frameworks were synthesized by Ni-catalyzed oxidative coupling without limitations on the substitution patterns. In addition, this method was also highly practical to the late-stage modification of bioactive molecules. Mechanistically, the use of acetone and a slight excess of triflate as terminal hydrogen acceptors was pivotal for the oxidative transformation. The combination of three distinct catalytic cycles, which contained two competitive oxidation processes and a Ni-catalyzed carbonyl Heck-type pathway, was proposed.

**Scheme 2:**
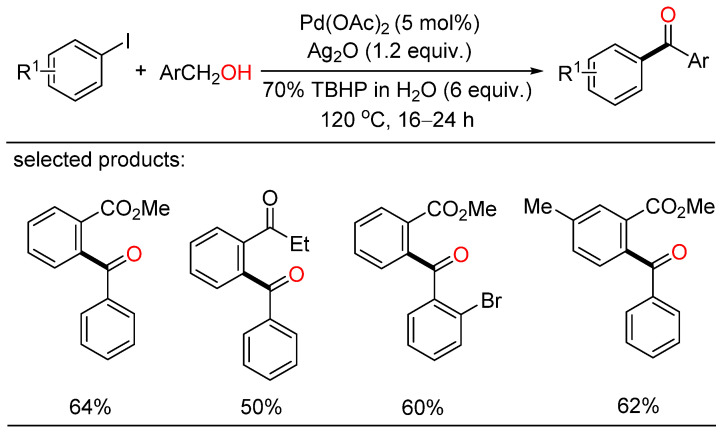
Palladium-catalyzed direct oxidative acylation of iodoarenes with primary alcohols.

Soon after, Satyanarayana reported palladium-catalyzed direct oxidative acylation of iodoarenes with primary alcohols, leading to the formation of various ketones (Scheme 2) [24]. In addition, this strategy was also applied to the synthesis of natural as well as pharmaceutical products. However, a large amount of tert-butyl hydroperoxide (TBHP) as the oxidizing agent was essential for this kind of oxidative coupling.

**Scheme 3:**
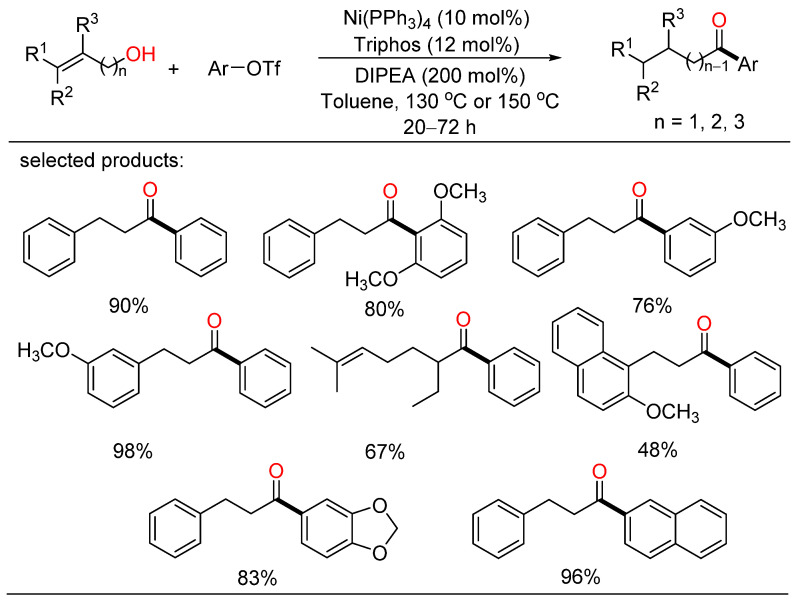
Redox-neutral cross-coupling of alkenyl primary alcohols and aryltriflates.

In 2021, Rong developed a redox-neutral cross-coupling approach for the construction of C-C bond with alkenyl primary alcohols and aryltriflates, providing a range of desired ketone products with good yields and broad functional group tolerance (Scheme 3) [25]. This method also afforded more functionalized ketones via the nickel/Triphos catalytic system without the addition of an external oxidant or reductant. A plausible catalytic cycle including isomerization of an alkenyl primary alcohol and a coupling process for the formation of a new C-C bond was given.

From a synthetic point of view, the direct conversion of C-H bonds into C-C bonds can potentially result in a more efficient synthesis with a reduced number of synthetic operations and thus be a more straightforward alternative in ketone synthesis.

**Scheme 4:**
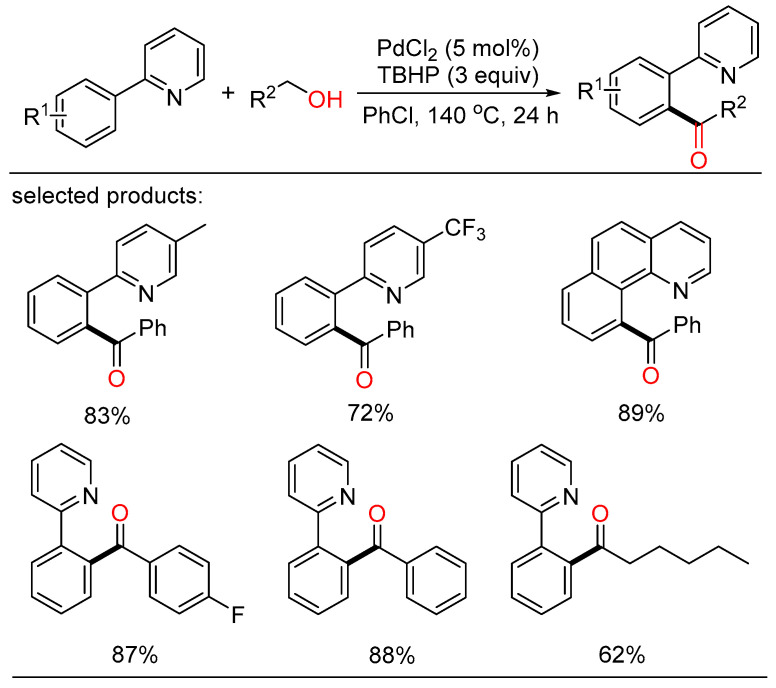
Palladium-catalyzed directing acylation of aryl C-H bonds.

According to this viewpoint, Deng described a palladium-catalyzed regio-selective acylation of aryl C-H bonds with the assistance of directing group (Scheme 4) [26]. Several benzylic and aliphatic alcohols were chosen as the economic acyl surrogates and were oxidized in situ in the presence of peroxide. Consequently, a series of aryl ketones were gained with good yield and high regioselectivity.

Compared to the above protocols which need a stoichiometric of peroxide as the oxidant, redox-triggered synthetic strategies have aroused much attention for their ability to expeditiously achieve complex ketones from simple alcohols, leading to a higher economical step to the classical synthetic routes.

**Scheme 5:**
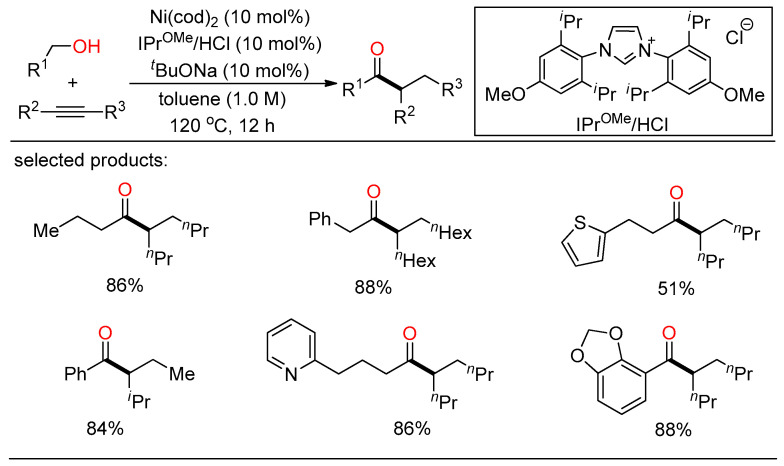
NHC/Ni-catalyzed hydroacylation of alkynes with alcohols.

In 2020, Shi demonstrated a selective NHC/Ni-catalyzed hydroacylation of alkynes with alcohols for the preparation of ketones (Scheme 5) [27]. A range of branched ketones were readily synthesized from various benzylic and aliphatic alcohols and internal alkynes in one step without the need for any oxidative or reductive additives.

**Scheme 6:**
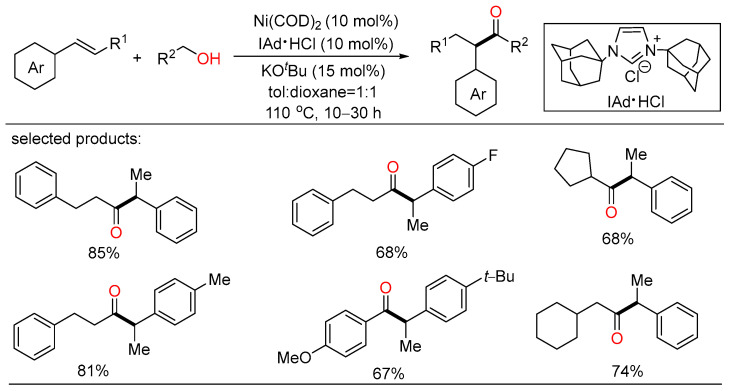
Ni-catalyzed dehydrogenative cross-coupling reaction cascade between alcohols and olefins.

**Scheme 7:**
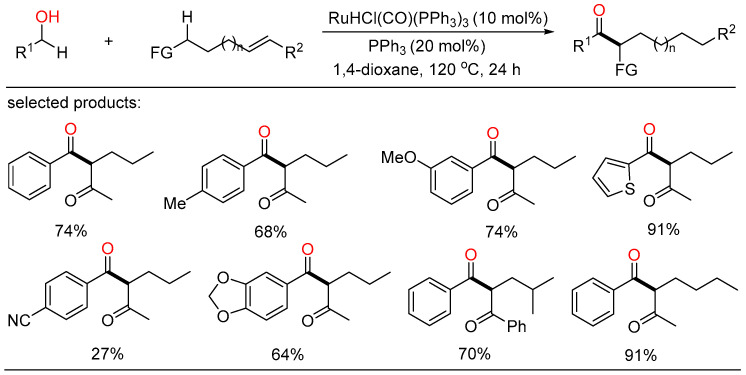
Remote C(sp^3^)-H functionalization of olefins with alcohols.

After a short while, Shu and Xia independently reported redox-triggered metal-catalyzed cross-coupling reactions from readily available alcohols and olefins. In Shu’s work, a variety of α-monoarylated ketones were selectively obtained by Ni-catalyzed dehydrogenative cross-coupling reaction cascade between alcohols and olefins (Scheme 6) [28]. This operationally simple procedure featured the utilization of commercially available and naturally abundant starting, allowing for the direct construction of monoarylated ketones with good yield, exclusive selectivity, and wide functional group tolerance. The nickel catalysts played a twofold role in two catalytic cycles. One cycle is for the oxidative dehydrogenation of alcohols to aldehydes, the other is for regioselective hydroacylation of aldehydes with olefins. On the other side, Xia disclosed a new method for the remote C(sp^3^)-H functionalization of olefins with alcohols (Scheme 7) [29]. The redox strategy was realized via a Ru-catalyzed α-acylation of olefins bearing a carbonyl group with primary alcohols, thus giving useful 1,3-dicarbonyl compounds without a base or an oxidant. Mechanistic investigations revealed that this step-economical transformation involved multiple processes, including borrowing hydrogen, hydrometallation, and metal walking.

**Scheme 8:**
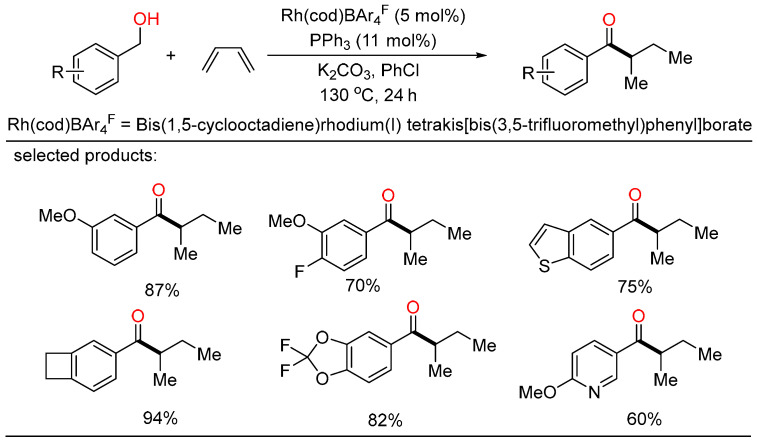
Rh-catalyzed redox-neutral coupling of benzylic alcohols with butadiene.

In 2021, the first Rh-catalyzed redox-neutral conversion of primary benzylic or aliphatic alcohols with butadiene was developed by Krische (Scheme 8) [30]. In the protocol, various isobutyl ketones containing electronically diverse aromatic and heteroaromatic rings were successfully obtained via merged transfer hydrogenative carbonyl addition-redox isomerization. Deuterium experiments demonstrated that the rhodium alkoxide species obtained upon carbonyl addition rendered redox isomerization without dissociation of rhodium at any stage. This elegant strategy contributed an alternative way that transformed inexpensive and abundant chemical feedstocks to value-added products under simple conditions.

**Scheme 9:**
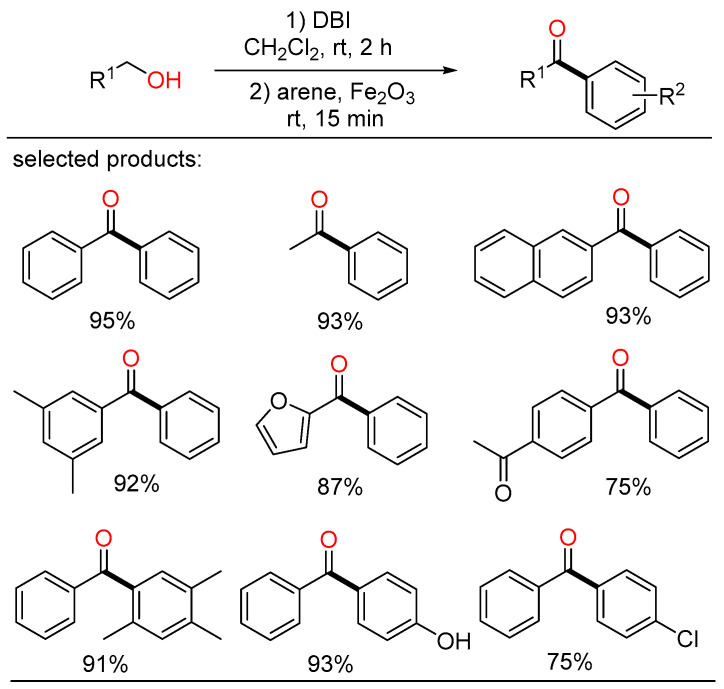
Cascade coupling of alcohols with arenes.

In the meantime, Kim displayed a novel method for the synthesis of various aryl ketones from primary alcohols at room temperature (Scheme 9) [31]. In this study, alcohols were converted into acyl bromide intermediates by the action of dibromoisocyanuric acid (DBI), followed by further transformation with arene compounds in the presence of Fe_2_O_3_ via Friedel–Crafts acylation to yield the corresponding ketone products in one pot.

## 3. C-O/C-S Bond Construction

Carboxylic acids and esters are prominent structural backbones that are widely existed in the synthesis of chemicals, drugs, materials, and polymers. Despite substantial well-established methodologies, the development of environmentally benign and cost-effective approaches for the construction of esters continues to attract great concern [32,33].

**Scheme 10:**
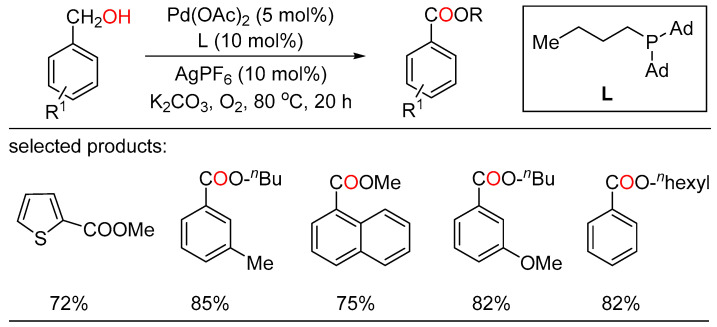
Palladium-catalyzed oxidative esterifications of benzylalcohols.

**Scheme 11:**
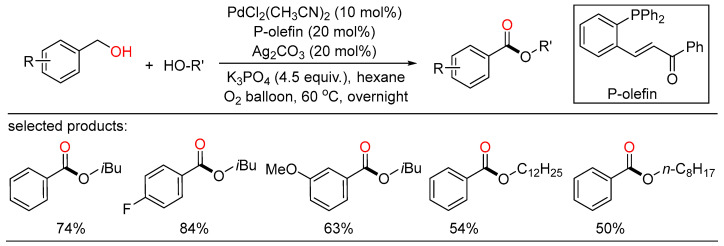
Palladium-catalyzed oxidative esterifications of benzylalcohols.

In 2010, Beller reported palladium-catalyzed oxidative esterifications of primary alcohols using dioxygen as benign oxidant, providing a general route for the synthesis of desired esters with high selectivity (Scheme 10) [34]. Both oxidative homocoupling reactions as well as cross-esterifications of benzyl alcohols with various aliphatic alcohols took place under mild conditions to give the corresponding esters with water as the only byproduct. In addition, these general green and selective oxidative conversions proceeded smoothly in the presence of simple catalytic systems without the aid of extra organic hydrogen acceptors. Simultaneously, Lei showed another Pd-catalyzed aerobic oxidative esterification of benzylic alcohols (Scheme 11) [35]. In this sustainable transformation, a range of aliphatic alcohols, including methanol and long-chain primary alcohols, successfully coupled with various benzylic alcohols to afford the target esters in moderate to high yields with good chemoselectivity. Both of these reaction pathways involving two Pd-catalyzed oxidation steps were outlined.

**Scheme 12:**
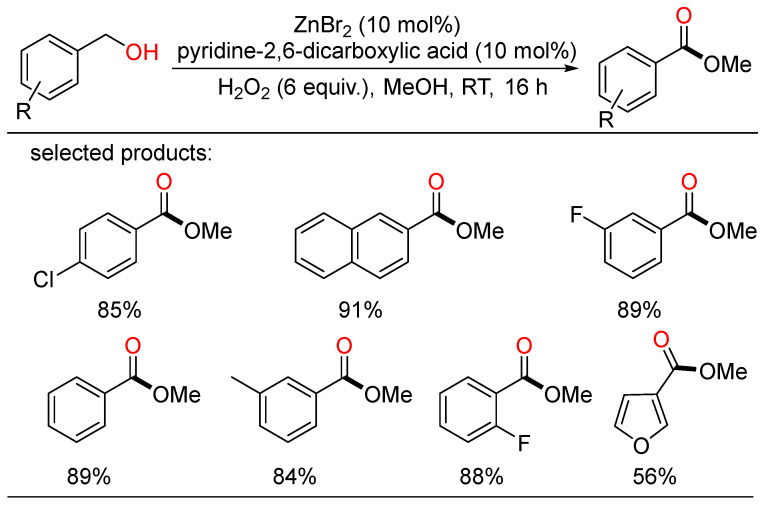
Zn-catalyzed oxidative esterifications of benzyl alcohols.

In 2012, Wu developed the first Zn-catalyzed oxidation of benzyl alcohols under air at room temperature using hydrogen peroxide as benign oxidant, providing a class of methyl benzoates in moderate to excellent yields (Scheme 12) [36].

**Scheme 13:**
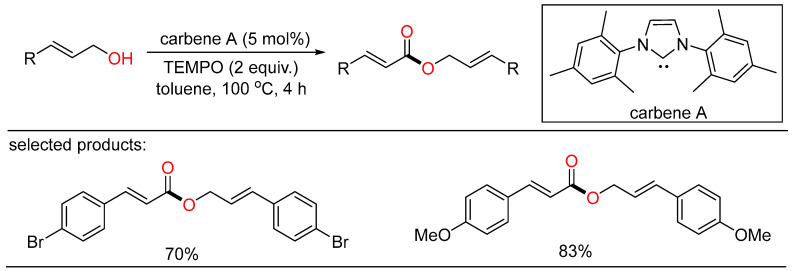
NHC-catalyzed oxidative esterifications of alcohols.

In 2013, Jang discovered NHC-catalyzed oxidative coupling of allylic alcohols. Some cinnamyl alcohols were subjected to the tandem oxidation/oxidative esterification system, giving α,β-unsaturated esters in moderate to good yields with the merger of stoichiometric TEMPO as the oxidant and methanol as the additive (Scheme 13) [37]. The author presumed that methanol might be helpful to the deprotonation of the α-proton of cinnamyl alcohol.

**Scheme 14:**
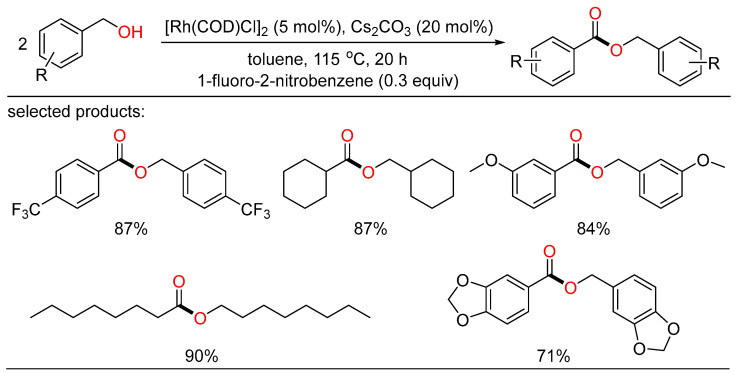
Rh-catalyzed oxidative homocoupling esterifications of alcohols.

Another homocoupling example of alcohols to esters was reported by Chung. They developed a robust Rh/base catalytic system that can be readily conducted to selectively yield the desired esters without ligand. A number of alcohols attached by various functionalities behaved well with nitroarene as the hydrogen acceptor (Scheme 14) [38].

**Scheme 15:**
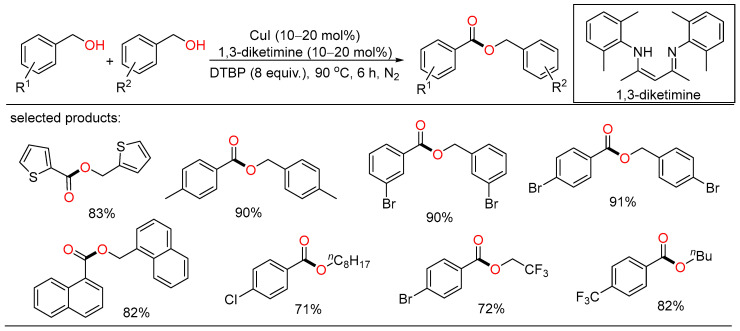
Cu-catalyzed oxidative esterifications of alcohols.

A solvent-free protocol for the copper-catalyzed selective oxidation of primary alcohols to esters was developed by Wei. Under neat conditions, homocoupling esterification and cross-esterification of benzylic alcohols with various aliphatic alcohols proceeded successfully to afford the target esters in good yields (Scheme 15) [39].

**Scheme 16:**
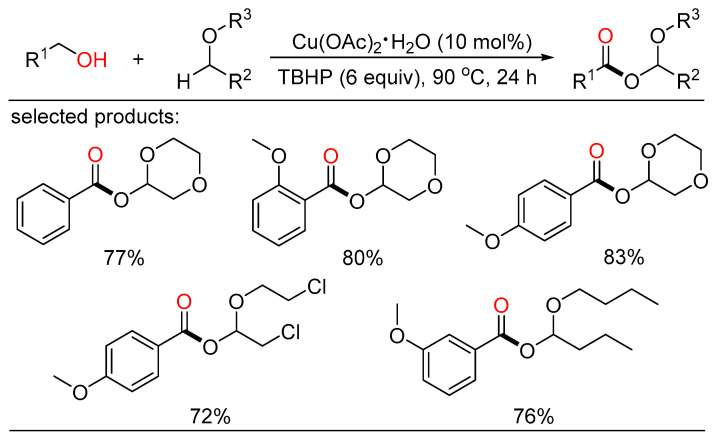
Cu-catalyzed oxidative esterifications of alcohols with ethers.

In 2014, Fu and Yuan jointly reported copper-catalyzed construction of C-O bonds through oxidative coupling of benzylic alcohols with ethers in open air (Scheme 16) [40]. A variety of α-acyloxy ethers were garnered in good yields with TBHP as the oxidant. A TBHP-initiated, copper-promoted radical mechanism for this oxidation reaction was proposed.

**Scheme 17:**
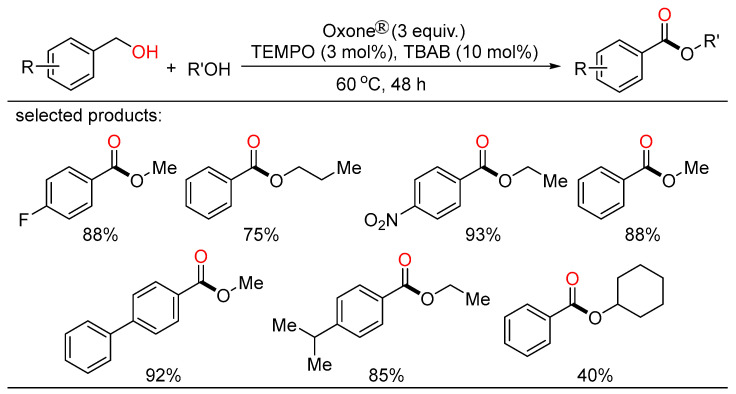
Oxone-mediated oxidative esterifications of alcohols.

Meanwhile, Maiti and Lahiri unveiled novel approaches towards selective cross- and self-oxidative esterification of a broad range of alcohols (Scheme 17) [41]. The cross-esterification occurred under a transition-metal-free condition with the combination of catalytic TEMPO/TBAB and Oxone^®^ as the oxidant, while the self-esterification was accomplished by the involvement of Fe(OAc)_2_/pyridine-2,6-dicarboxylic acid as the active catalyst.

**Scheme 18:**
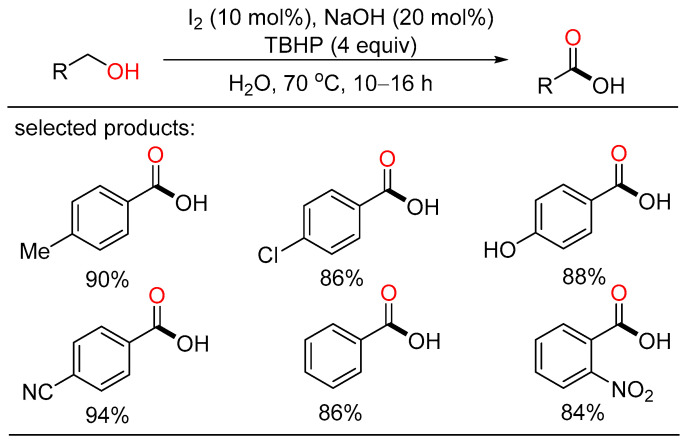
I_2_-catalyzed oxidative carboxylation of alcohols.

In 2017, Elias described I_2_/NaOH-catalyzed oxidation of alcohols for the preparation of carboxyl acids using water as the solvent (Scheme 18) [42]. A series of aryl- and heterocycle-substituted carboxyl acids were efficiently obtained with good yield under mild reaction conditions. Mechanistic studies indicated that IO_2_^−^ is the most probably active catalyst which was formed and regenerated by the oxidation of IO^−^ by TBHP. This metal-free protocol did not require chromatographic purification, featuring operational convenience, broad substrate scope, and easy scalability.

**Scheme 19:**
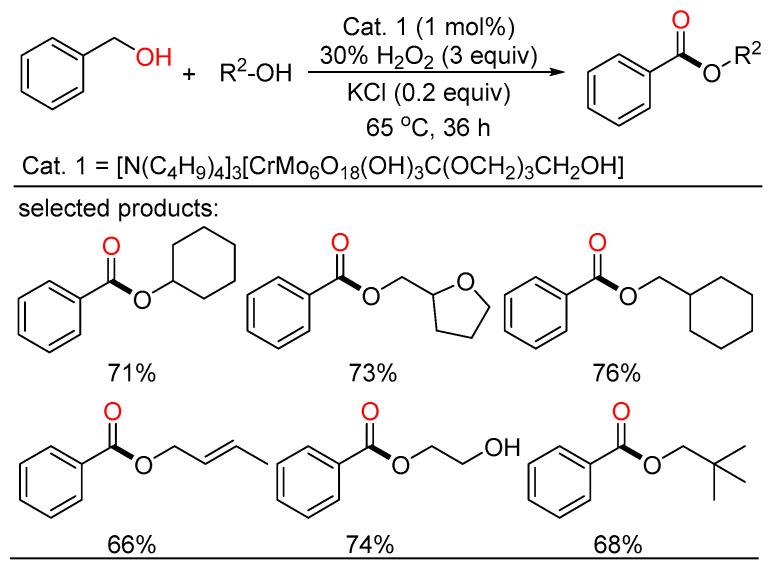
Chrome-catalyzed oxidative esterifications of alcohols.

Later, Wei reported a novel Anderson-type chrome-based catalyst which can render the direct transformation from alcohols to esters by H_2_O_2_ oxidation in good yields and high selectivity without extra ligands (Scheme 19) [43]. A variety of alcohols with different functionalities including some natural products and pharmaceutical intermediates are tolerated in this system. The catalyst can be applied to gram-scale reactions, and retain good catalytic reactivity after recycling several times, indicating potential value of the catalytic system in industrial realm.

**Scheme 20:**
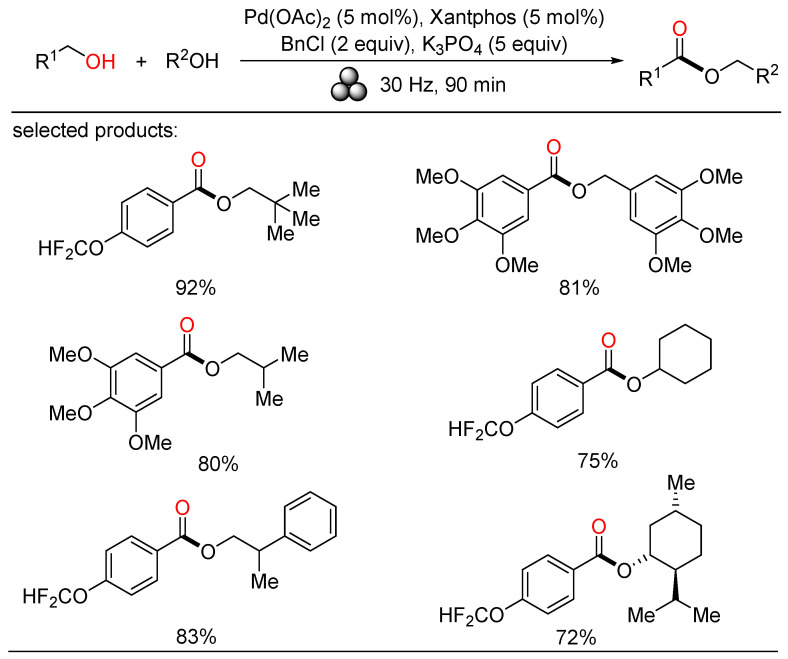
Pd-catalyzed mechanochemical oxidative esterifications of alcohols.

In 2022, Bolm exploited a mechanochemical procedure for the palladium-catalyzed oxidative esterification of alcohols, allowing a solvent-free synthesis of symmetrical and unsymmetrical esters in up to excellent yields after short reaction times at ambient temperature (Scheme 20) [44]. Manifold liquid and solid benzylic and aliphatic alcohols were easily self-esterified and cross-esterified under such mild reaction conditions. Moreover, secondary alcohols were also carried out in the cross-esterification with benzylic alcohols. This sustainable protocol enabled significant waste reduction by evaluating some metrics, providing a complement to solution-based methods.

**Scheme 21:**
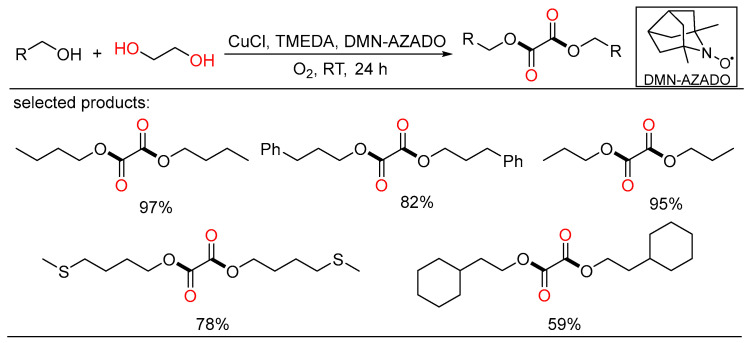
Cu-catalyzed oxidative diesterifications of alcohols.

At the same time, Yamaguchi showed a highly selective method for the selective aerobic oxidation of aliphatic primary alcohols, demonstrating the formation of oxalic acid diesters via oxidative esterification from alcohols and ethylene glycol using the CuCl/TMEDA/DMN-AZADO catalytic system (Scheme 21) [45]. This novel project was fulfilled under mild reaction conditions using dioxygen as the terminal oxidant and only required a stoichiometric ratio of alcohols and ethylene glycol. Detailed experiments and DFT calculations were conducted to elucidate the mechanism of the novel oxidation reactions.

**Scheme 22:**
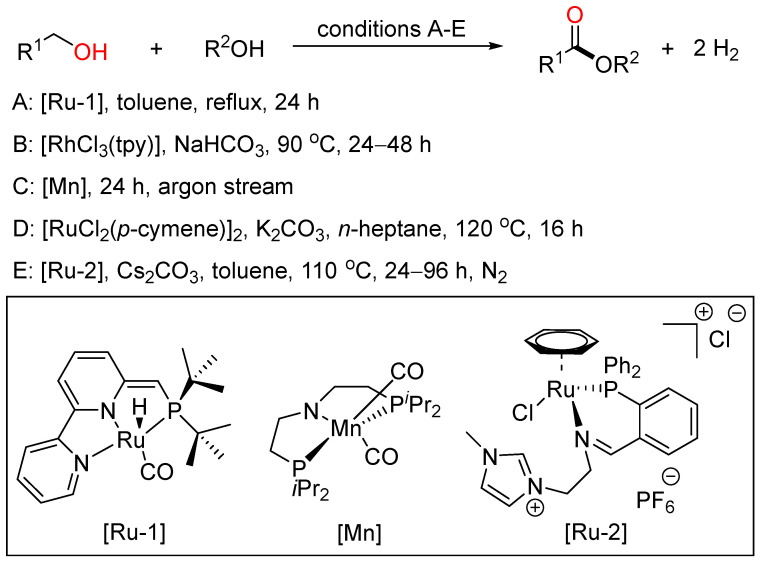
Transition metal-catalyzed dehydrogenative esterifications of alcohols.

Although the oxidative coupling reactions of alcohols provide efficient patterns to acquire esters, from the viewpoint of sustainable chemistry, the acceptorless dehydrogenative coupling (ADC) reaction of alcohols would be the most promising method for ester synthesis because it appears as an atom- and step-economical process accompanied by the extrusion of gaseous hydrogen as the only byproduct (Scheme 22).

In 2012, Milstein employed a bipyridyl-based Ru(II) pincer catalyst to realize cross-dehydrogenative coupling of primary alcohols with secondary alcohols, affording esters with the liberation of dihydrogen in high yield and good selectivity under neutral conditions (**A**) [46]. In 2016, Xiao reported Rh-terpyridine catalyzed dehydrogenative cross-coupling of primary alcohols to yield esters with broad substrate scope, good functional group tolerance and high selectivity (**B**) [47]. Then, Gauvin described aliphatic pincer-supported earth-abundant manganese complexes as effective catalysts for the acceptorless dehydrogenative coupling of a wide range of alcohols to esters under base-free conditions. The reaction proceeded smoothly under neat conditions, with modest catalyst loading and releasing only dihydrogen as byproduct (**C**) [48]. Then, Zhao and Li cooperatively developed a Ru-catalyzed highly selective dehydrogenative cross-coupling of alcohols to esters using phosphine oxide containing ligands based on the aldehyde effect (**D**) [49]. Recently, Fu and Li together presented the acceptorless dehydrogenative cross-coupling of primary alcohols to access esters effected by designing a novel Ru complex as the catalyst. These two sustainable protocols were tolerated to a wide range of primary alcohols to give various unsymmetrical esters with good functional group compatibility and high selectivity (**E**) [50].

**Scheme 23:**
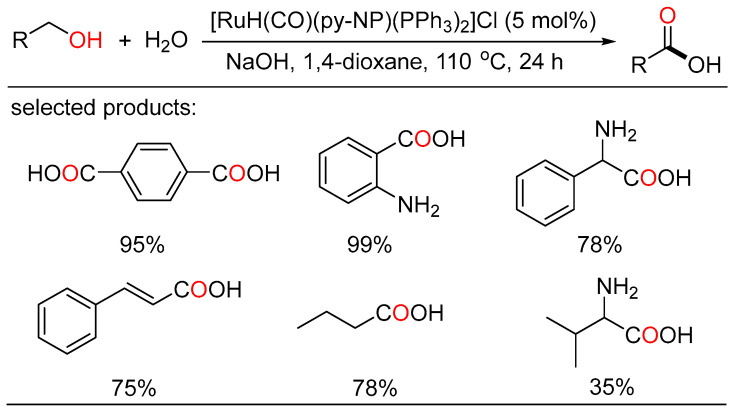
Ru-catalyzed dehydrogenative carboxylation of alcohols.

**Scheme 24:**
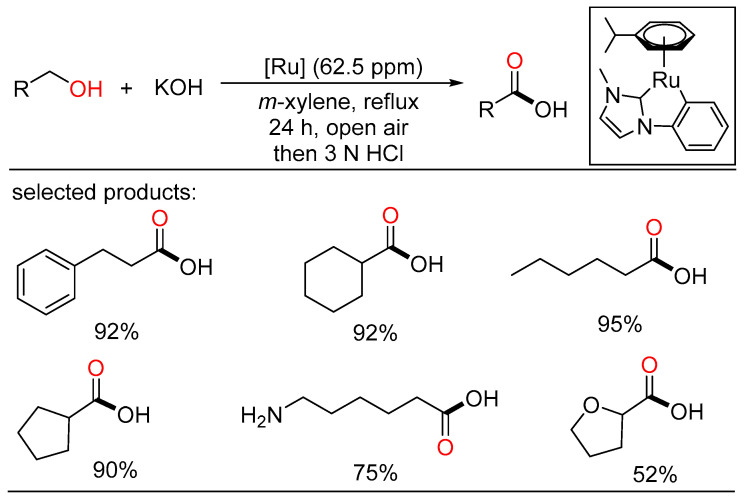
Ru-catalyzed dehydrogenative carboxylation of alcohols.

The ADC strategy was also applied in the synthesis of carboxylic acid. Bera and Verpoort reported Ru-catalyzed direct conversion of alcohols to carboxylic acids under basic conditions, respectively. In the former method, a one-pot transformation of alcohols to carboxylic acids proceeded using alkaline water (Scheme 23) [51]. Various primary alcohols such as benzyl alcohols, long-chain alcohols, amino alcohols, and diols were tolerated in the catalytic system. Mechanistic studies indicated two sequential processes, namely acceptorless dehydrogenation of alcohols to aldehydes, and following “aldehyde-water shift” reaction. In the latter work, Verpoort et al. developed a graceful method of acceptorless dehydrogenative coupling of alcohols with hydroxides by orchestrating a series of NHC-Ru complexes (Scheme 24) [52]. Then, they evaluated the effects of several substituents on the catalytic performance of the resulting NHC-Ru complexes. Gram-scale preparation of carboxylic acids was also conducted at an ultralow catalyst loading in open air.

**Scheme 25:**
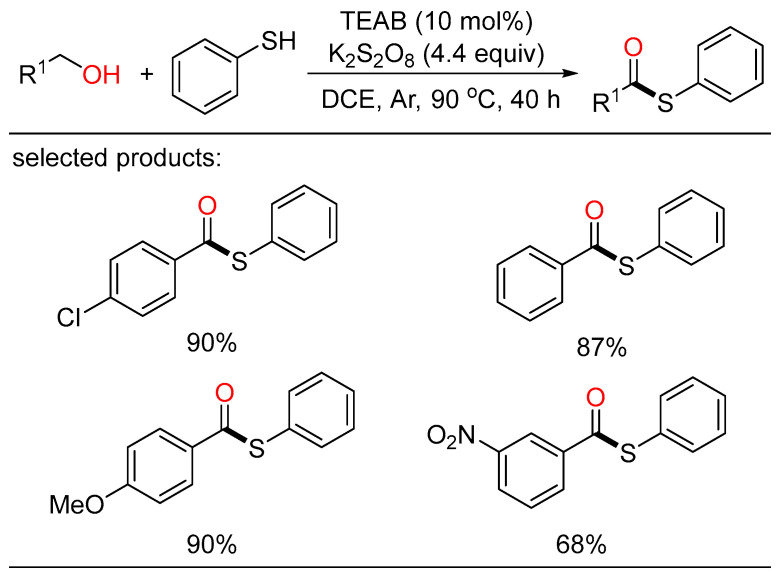
TEAB-catalyzed oxidative coupling of alcohols with thiophenols.

As a family of activated carboxylic acid derivatives, thioesters are very versatile building blocks in the fields of chemistry and biology. Based on the importance of these compounds, Zhu disclosed a tetraethylammonium bromide (TEAB)-catalyzed oxidative coupling of alcohols with thiophenols or disulfides (Scheme 25) [53]. This metal-free protocol offered a convenient route to the formation of a wide range of thioesters with broad substrate scope in high yields. Mechanistically, cross-coupling of the acyl- and sulfur radicals generated in the oxidizing environment resulted in the formation of the corresponding thioesters.

## 4. C-N Bond Construction

Amide functional groups represent one of the most salient structures in organic chemistry and find tremendous usage in many areas, such as biological molecules, natural products, pharmaceutical drugs, and polymer materials [54]. The classical procedures for the synthesis of amides depend on condensations of amines with carboxylic acids and derivatives [55]. Despite being convenient, these approaches need stoichiometric activators and commonly cause a large amount of waste production. Therefore, step- and atom-economical cross-coupling reactions undoubtedly provide a straightforward and environmentally friendly strategy for the construction of these highly useful skeletons.

Transition-metal-catalyzed amidation of alcohols with amines has been considered as a direct methodology that avoids the treatment of carboxylic derivatives, offering an appealing alternative to conventional acylation-based coupling reactions. Over the past few years, several efficient catalytic systems have been well developed and have become a powerful tool for amide bond formation [56,57,58,59].

**Scheme 26:**
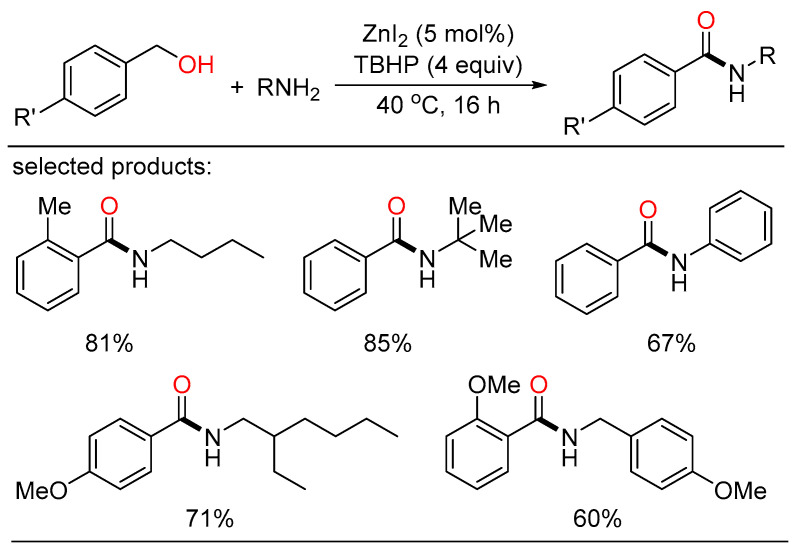
Zn-catalyzed oxidative amidation of benzyl alcohols.

In 2013, Beller and Wu cooperatively showed the first Zn-catalyzed oxidative amidation of benzyl alcohols with both aliphatic and aromatic amines with TBHP as the oxidant (Scheme 26) [60]. Under neat conditions, various benzamides, including heterocycles and secondary amides, were acquired in good to excellent yields.

**Scheme 27:**
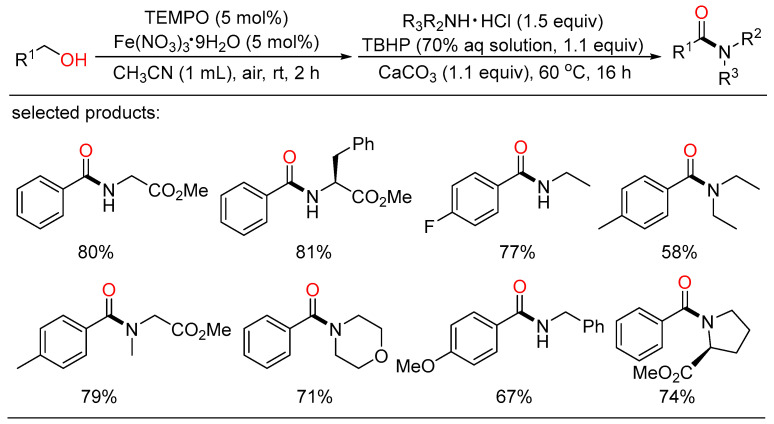
Fe-catalyzed cascade oxidative amidation of benzyl alcohols.

In the same year, Chen reported a tandem oxidative amidation of benzyl alcohols with amine hydrochloride salts using inexpensive iron-TEMPO catalytic system, air and TBHP as the oxidants (Scheme 27) [61]. Both secondary and tertiary benzamides could be prepared under mild conditions. A free radical mechanism of oxidative coupling pathway was demonstrated by the addition of a radical inhibitor, which led to total inhibition of amide formation.

**Scheme 28:**
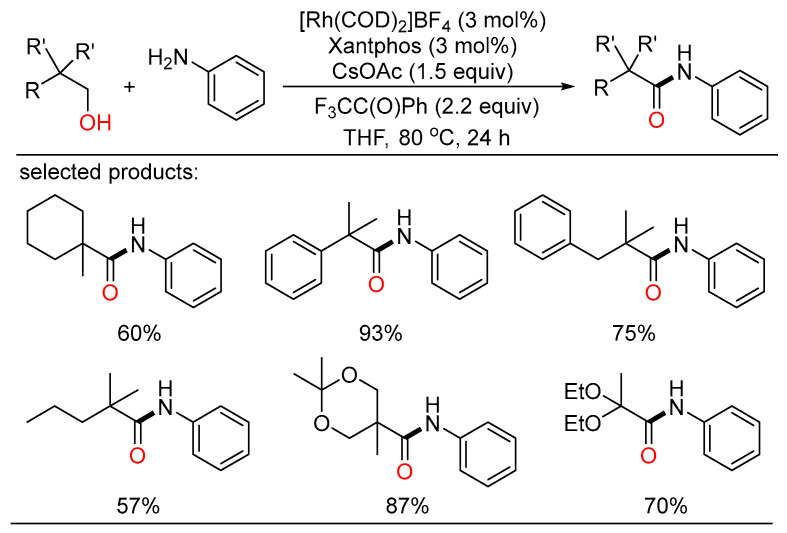
Rh-catalyzed cascade amidation of alcohols.

Nonetheless, amide synthetic methods based on sterically hindered alcohols were less explored because of their low reactivity. In 2016, Hull developed a rhodium catalytic system to realize the oxidative amidation reaction of sterically hindered alcohols to access amides (Scheme 28) [62]. A series of amine nucleophiles, both aliphatic and aromatic amines, were subjected and provided the corresponding amides containing a quaternary carbon in good to excellent yields. Mechanistically, an aldehyde-bound rhodium intermediate was a key species in the oxidation of alcohols.

**Scheme 29:**
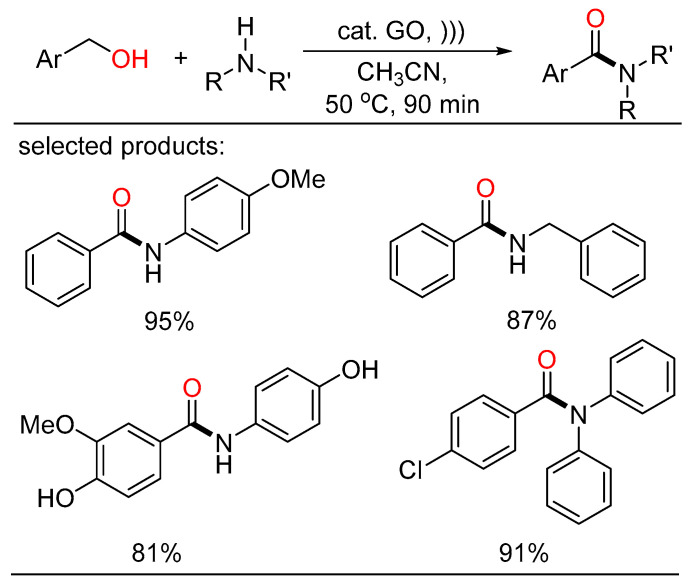
Graphite oxide-catalyzed cascade amidation of alcohols.

Ultrasound irradiation was also successfully applied in the direct oxidative amidation of benzyl alcohols with amines using graphite oxide (GO) as an oxidative and reusable solid acid catalyst. The oxidative amidation of benzyl alcohols went through in short reaction times under metal-free conditions, giving the corresponding amides in good to high yields (Scheme 29) [63].

**Scheme 30:**
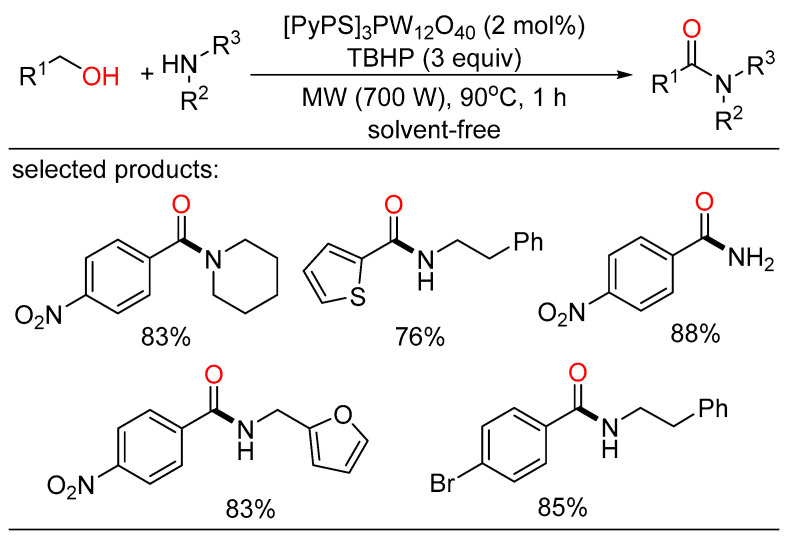
Ionic liquids-catalyzed cascade amidation of alcohols.

In the same year, an efficient protocol for the tandem oxidative amidation of alcohols with amines was unfolded by Yang and Yi. Using heteropolyanion-based ionic liquids as the catalyst and tert-butyl hydroperoxide as the oxidant, the amidation reaction afforded a variety of primary, secondary, and tertiary amides in moderate to good yields, highlighting solvent-free and microwave conditions, as well as renewability of catalysts (Scheme 30) [64].

**Scheme 31:**
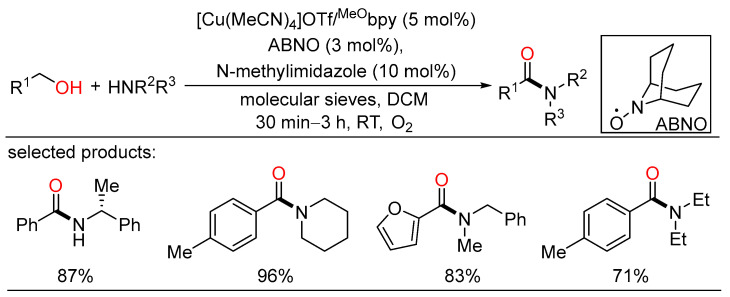
Cu-catalyzed amidation of benzyl alcohols.

**Scheme 32:**
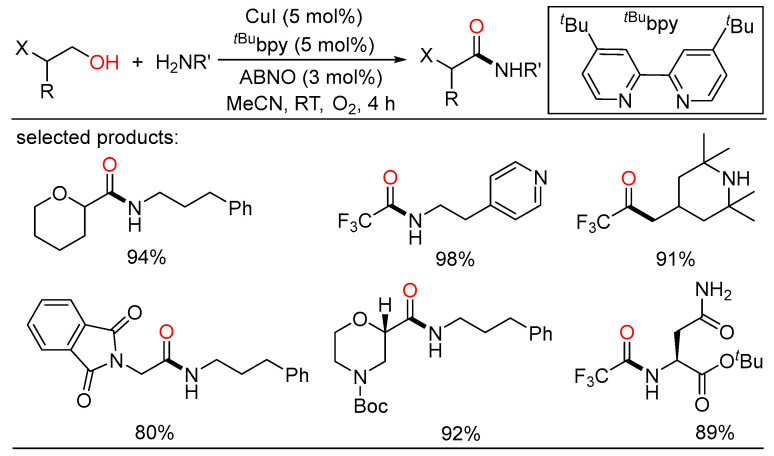
Cu-catalyzed amidation of alcohols.

Copper-catalyzed aerobic systems have also been widely applied in oxidative couplings of alcohols due to high efficiency and low price of copper catalysts. In 2016, Stahl described a robust Cu/nitroxyl catalyst system that enabled efficient aerobic oxidative coupling of alcohols and amines to amides at room temperature in short reaction times, exhibiting mild conditions and high catalytic performance (Scheme 31) [65]. Various benzylic alcohols and primary/secondary amines can be cross-coupled in this reaction, providing broad access to a wide range of secondary and tertiary amides. Following this, the same group used the analogous catalyst system to realize aerobic oxidative coupling of heteroatom-substituted primary alcohols and amines for the synthesis of diverse pharmaceutically relevant heteroatom-substituted amides (Scheme 32) [66]. Commercially available feedstocks, large scale experiments, mild reaction conditions, and broad substrate scope suggested potential utility of this elegant protocol in the area of medicinal chemistry. The reaction mechanism could be illustrated as follows: The oxidative amidation began with alcohol oxidation to the aldehyde, followed by coupling by an amine to give a hemiaminal intermediate, which was further oxidized to the corresponding amide.

**Scheme 33:**
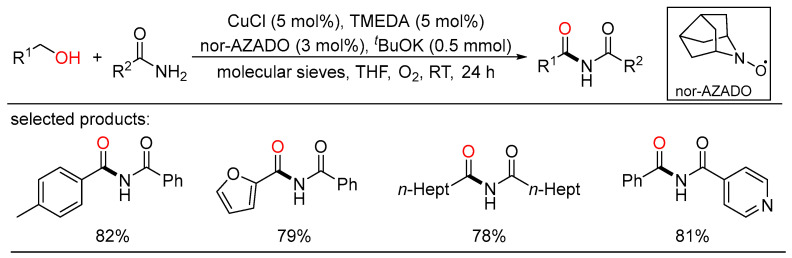
Cu-catalyzed amidation of alcohols with amides.

**Scheme 34:**
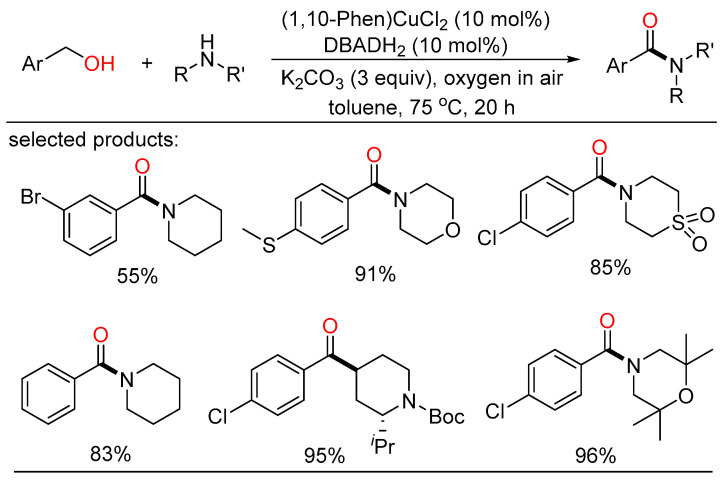
Cu-catalyzed aerobic amidation of alcohols.

By applying the similar Cu/nitroxyl catalyst system, Yamaguchi developed the first oxidative acylation of amides with alcohols to get structurally miscellaneous imides using dioxygen as the terminal oxidant (Scheme 33) [67]. This powerful strategy was also employed to synthesize α-ketocarbonyl derivatives from 1,2-diols and multiple nucleophiles, such as amides, amines, and alcohols. Detailed experiments revealed that the easily accessible TMEDA ligand was key to this aerobic oxidative acylation reaction. Moreover, Krabbe demonstrated a copper-catalyzed aerobic oxidative amidation for the direct conversion of (hetero)aryl primary alcohols to tertiary amides with a big number of secondary amines (Scheme 34) [68]. The convenience procedure harnessed readily available nonprecious copper catalyst and oxygen in air as the terminal oxidant. The conjunction of Cu(phen)Cl_2_, di-tert-butyl hydrazine dicarboxylate (DBADH_2_), and inorganic base ensured the production of assorted benzamides in moderate to excellent yields.

**Scheme 35:**
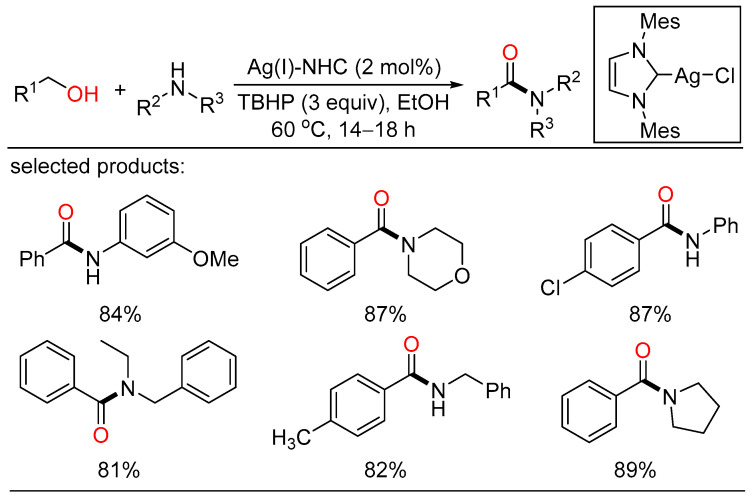
Ag-catalyzed amidation of alcohols.

In 2019, a silver/NHC-catalyzed oxidative coupling of alcohols and amines was unlocked for the first time. The amide compounds were smoothly generated with the help of TBHP as the oxidant and ethanol as the solvent. Control experiments were also carried out, proposing that the corresponding aldehyde and hemiaminal were the key intermediate for this tandem oxidation process (Scheme 35) [69].

**Scheme 36:**
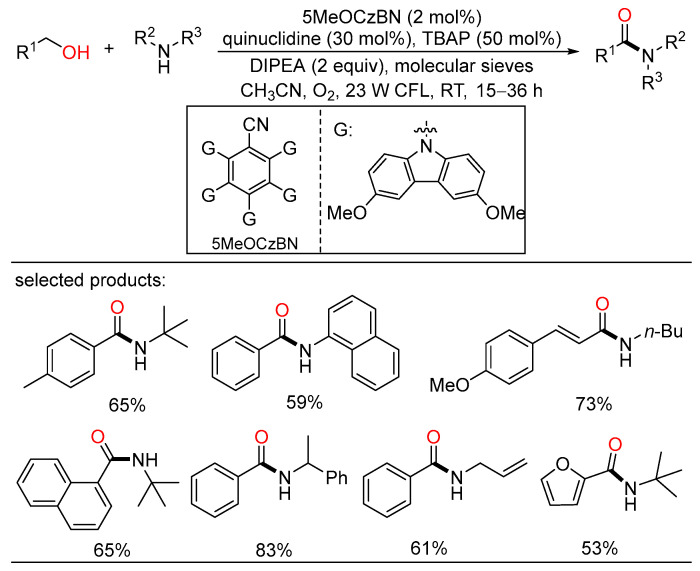
Photoredox oxidative amidation of alcohols.

In recent years, photoredox catalysis has emerged as a powerful and outstanding method in organic synthesis, allowing the innovation of sustainable and efficient procedures [70,71,72]. Based on the importance, an organophotoredox aerobic process for the construction of amide bonds by coupling alcohols and amines was reported by Singh in 2019. The combination of organic photocatalyst and quinuclidine (HAT catalyst) was essential for this kind of amides synthesis. Mechanistic research showed that the reduction of imine, generated from alcohol and amine, by the photocatalyst yielded amides. Various benzylic and aliphatic alcohols were successfully treated with alkyl and aryl amines, giving the amide products with good functional group tolerance at room temperature by the irradiation of compact fluorescent light (CFL) (Scheme 36) [73].

**Scheme 37:**
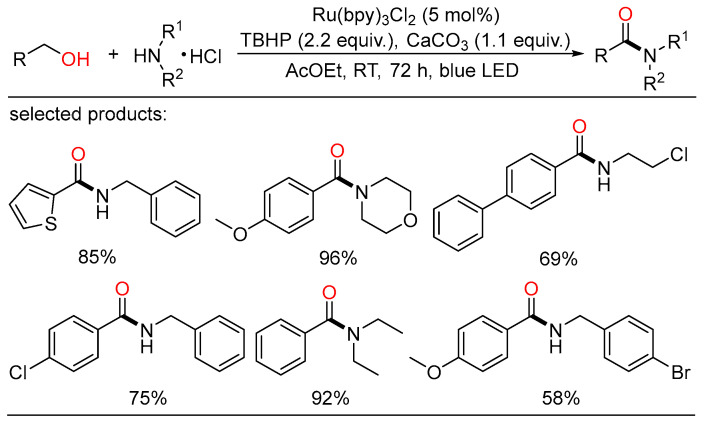
Photoredox oxidative amidation of alcohols.

In 2020, De Luca showed another visible-light-mediated photocatalyzed amide synthesis from alcohols and amines using ethyl acetate as an inexpensive solvent. An array of substituted alcohols and amine hydrochloride salts were tested to afford the corresponding amides in good yields with blue LED. This approach provided a novel complement to the classical methods with sustainability and applicability features (Scheme 37) [74].

**Scheme 38:**
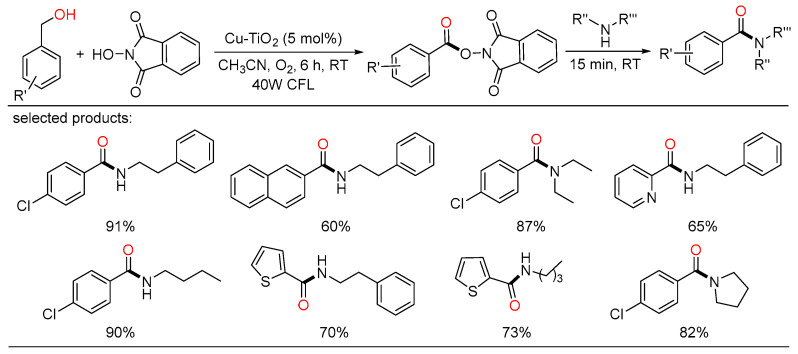
Cu-TiO_2_-based photoredox oxidative amidation of alcohols.

Several months later, a recyclable Cu-N-TiO_2_ heterogeneous photocatalyst enabled amides formation between alcohols and amines with the assistance of N-hydroxyphthalimide was described. Under the irradiation of a 40 W CFL, various amides were synthesized in short reaction time in high yields. This method had wide substrate scope and good functional group tolerance, and was also successfully applied to the drug synthesis on a gram scale (Scheme 38) [75].

Undoubtedly, the direct dehydrogenative coupling of alcohols and amines to afford amides was also a more sustainable and economical synthetic procedure [76,77,78]. Inspired by the pioneering work of Milstein in 2007 [79], a large stock of advancements in this field were exploited by many researchers. Herein, we summarized recent dehydrogenative coupling examples of alcohols and amines promoted by highly efficient metal-based catalytic systems.

Ruthenium was the most widely used catalyst for the construction of amides via direct dehydrogenative coupling of alcohols and amine. Over the past few years, a batch of Ru-based catalytic systems was developed for amides synthetic strategy [80,81,82,83,84,85,86,87,88,89,90,91,92,93,94,95,96]. Notwithstanding, most of these cases often confronted harsh reaction conditions (reflux temperature), limited substrate scope (primary amines), and pre-prepared complex catalysts. Thus, there is much space to unlock more active Ru-based catalytic systems for less reactive aryl and secondary amines under milder conditions.

**Scheme 39:**
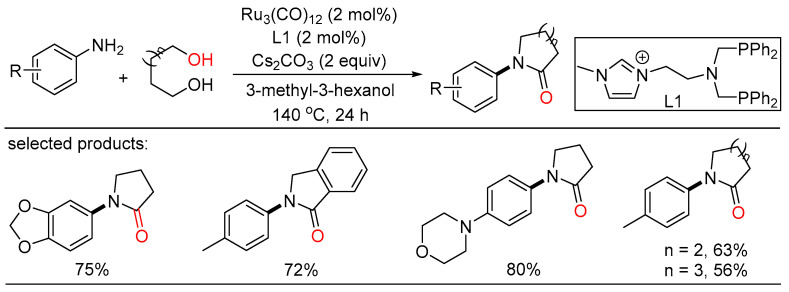
Ru-catalyzed dehydrogenative amidation of alcohols.

In 2019, Li and Fu together showcased the first example of N-substituted lactams synthesis via acceptorless dehydrogenation coupling of diols with primary amines in one step, which was rendered by merging Ru_3_(CO)_12_ with a hybrid NHC-phosphine-phosphine ligand as the optimal catalyst (Scheme 39) [97]. Detailed experiments indicated that cascade N-alkylation and amidation processes were involved to yield the final products.

**Scheme 40:**
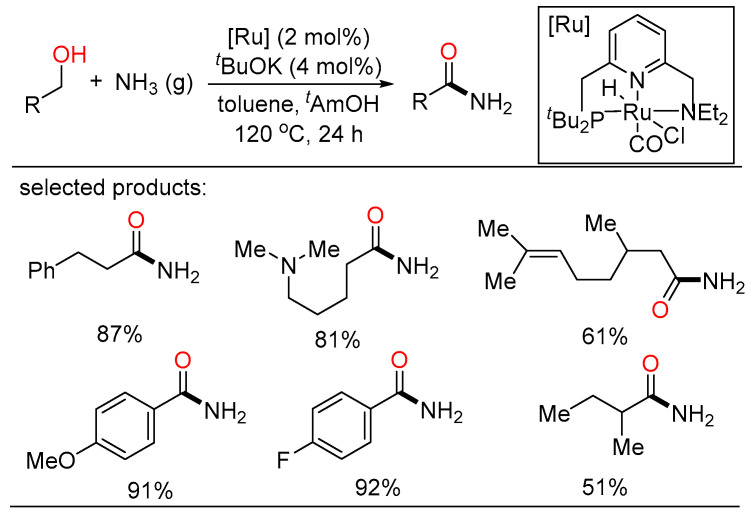
Ru-catalyzed dehydrogenative amidation of alcohols with ammonia.

Ammonia is the simplest, most abundant starting material for the industrial preparation of nitrogen-containing compounds. Accordingly, Milstein developed the unprecedented a selective ruthenium-catalyzed preparation of primary amides from alcohols and ammonia in 2022. Various aliphatic and aromatic primary amides were synthesized in high yields without observing other N-containing byproducts. Control experiments and DFT calculations demonstrated the selectivity to both kinetic and thermodynamic preference for the formation of desired primary amides over other N-containing compounds (Scheme 40) [98].

**Scheme 41:**
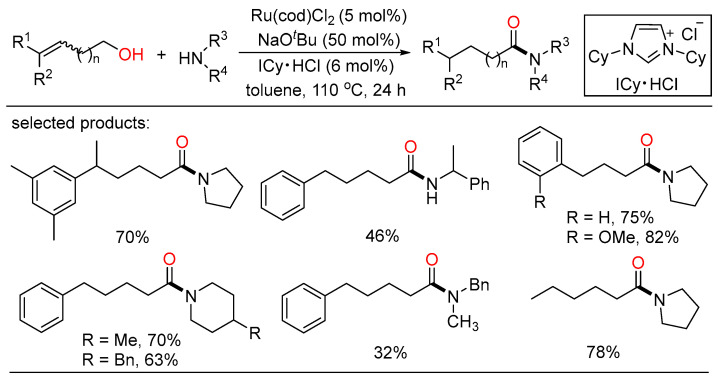
Ru-catalyzed chain-walking redox-neutral amidation of alcohols.

Transition-metal-catalyzed alkene isomerization by “chain-walking” strategy in combination followed by a successive transformation could realize remote site-selective functionalization, which can reduce manipulated steps and functional group protection/deprotection processes, and has stimulated considerable interest and exploration [99,100]. However, related remote amidation reactions with amines were less discovered. Lately, Rong and co-workers depicted a simple redox-neutral ruthenium catalytic system for the isomerization of long-range alkenyl alcohol with amines to furnish the corresponding amide products (Scheme 41) [101]. A wide range of functional groups as well as biologically active molecules were well tolerated in different substituted alkenyl alcohols and amines. This exclusive redox-neutral tandem system with a high atom and step economy displayed great potential for the selective construction of pharmaceutical units and complex structures.

**Scheme 42:**
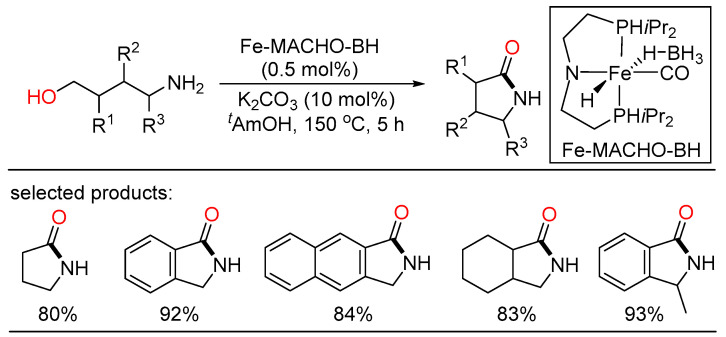
Fe(II) pincer-catalyzed dehydrogenative lactam formation from amino alcohols.

Moreover, the development of synthetic procedures that obviate the use of noble metals was highly demanding. Other nonprecious metals, such as Fe, Ni, and Mn, were also practical and effective for the dehydrogenative coupling synthesis of amides. In 2014, Beller introduced an iron(II) pincer-catalyzed dehydrogenative methodology for the synthesis of lactams from amino alcohols through a versatile domino sequence (Scheme 42) [102]. This tandem process happened through initial dehydrogenation of the substrates, subsequent intramolecular cyclization, and final oxidation to generate the target products in good yields. The ability to get heterocycles of different sizes render this protocol applicable and versatile, in which two sequential oxidation processes were took place without demanding an external oxidant. In 2017, Bernskoetter employed the same iron complex for the dehydrogenative intermolecular coupling of alcohols and secondary amines to give tertiary amides with high turnover numbers up to 600 [103]. This iron-promoted amidation offered preference for secondary amines, which was an appealing alternative to the previous precious-metal catalysts, which most performed well with primary amines. However, the main drawback of this iron catalytic system was the steric limitation of substrate scope.

**Scheme 43:**
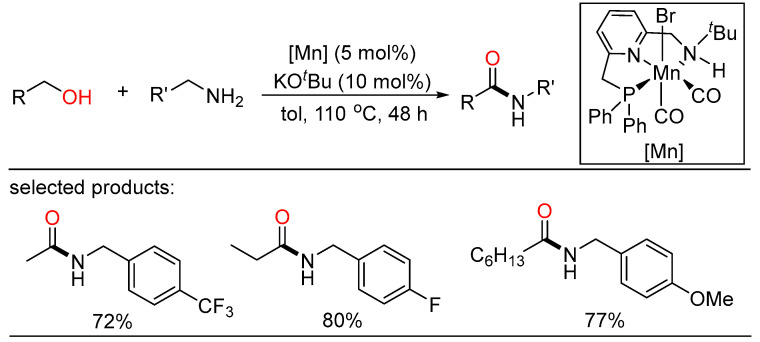
Mn(I) pincer-catalyzed amidation of alcohols.

In 2017, Milstein reported the first example for amides synthesis via dehydrogenative coupling of amines with alcohols using manganese pincer complex as catalyst, highlighting atom-economical and sustainable (Scheme 43) [104]. In this method, a manganese complex bound to the hemiaminal was considered to be the key intermediate.

**Scheme 44:**
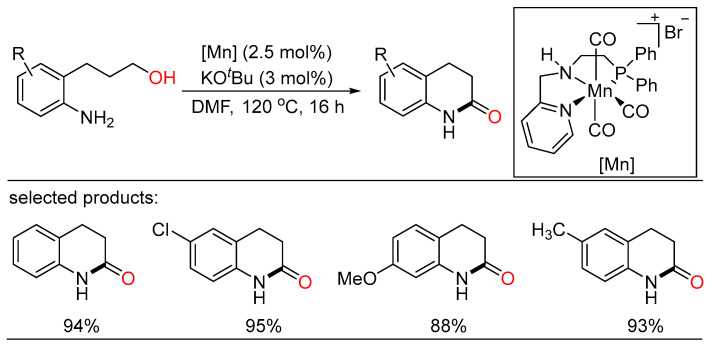
Mn(I) pincer-catalyzed lactam formation from amino alcohols.

In 2022, Rueping developed a novel manganese-catalyzed dehydrogenative and redox-neutral heterocyclization of amino alcohols, providing a range of N-containing heterocycles, especially quinoline derivatives, in good to excellent yields. The intramolecular reaction was carried out under facile conditions using earth-abundant, air and moisture stable manganese catalysts (Scheme 44) [105].

**Scheme 45:**
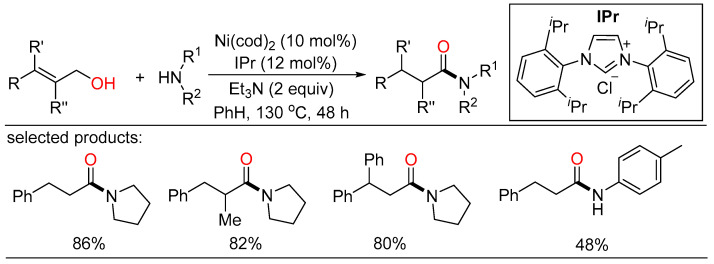
Ni/NHC-catalyzed redox-neutral amidation of allyl alcohols.

In the meantime, Rong described a Ni/NHC-catalyzed redox-neutral dehydrogenative cross-coupling reaction of alcohols and amines (Scheme 45) [106]. This methodology featured base metal catalysis, commercially available catalyst, avoidance of oxidant, good reaction yields, wide functional group tolerance and substrate scope, which showed potential application of this synthetic procedure. Moreover, the mechanism of the reaction was demonstrated by NMR spectroscopy and isolation of intermediates.

It is worth noting that amines are commonly obtained from the corresponding nitro or nitrile compounds requiring extra excess reducing agents. In this regard, although transition-metal-catalyzed dehydrogenative coupling between alcohols and amines provides an efficient and versatile way to amides with high atom economy and waste prevention, it is highly desirable to develop convenient methodologies to utilize stable, cheap, and easily handled nitro or nitrile compounds as amino precursors instead of amines to realize amides preparation.

**Scheme 46:**
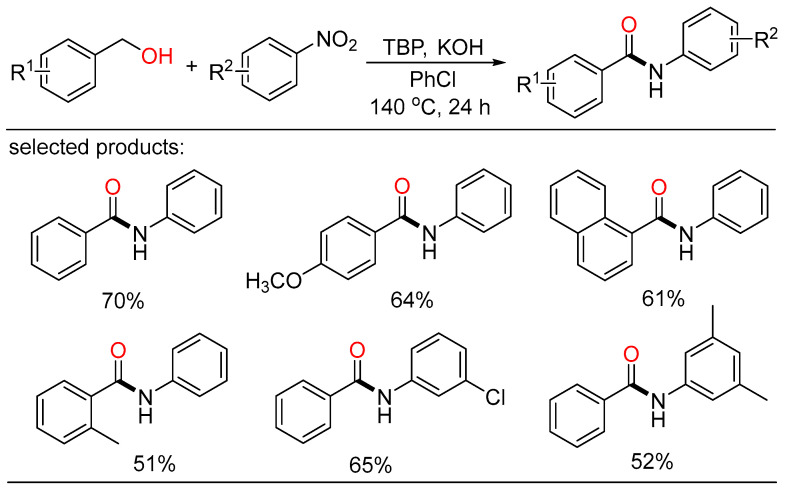
Peroxide-mediated amidation of alcohols with nitroarenes.

To this end, Deng described a novel and direct amidation of alcohols with nitroarenes mediated by peroxide (Scheme 46) [107]. Aromatic amides were achieved in moderate to good yields under transition-metal-free conditions. The reaction showed very good selectivity with amides as the major products. The peroxides and solvents gave a significant influence on the reaction yield. Hydrogen generated from alcohol oxidation acted as the reducing agent. The nitro reduction, alcohol oxidation, and amide formation were accomplished in one step.

**Scheme 47:**
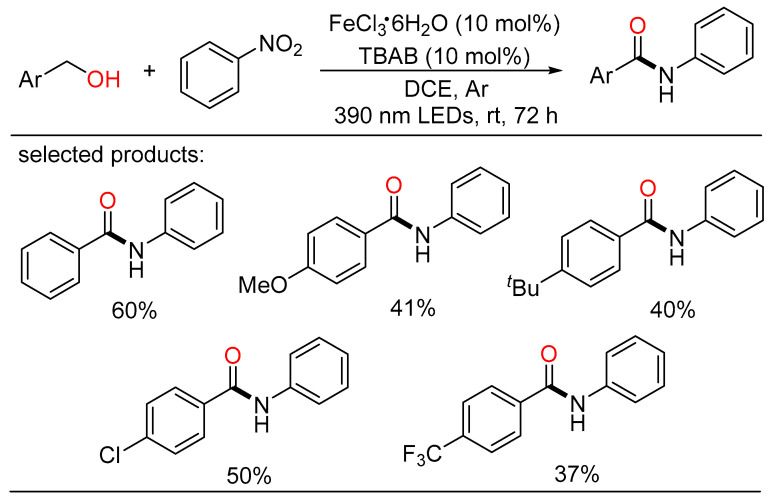
Fe-catalyzed amidation of alcohols with nitroarenes.

Following this, Zeng and Li cooperatively reported a photoredox-triggered amide preparation of late (Scheme 47) [108]. By employing readily available nitroarenes as the nitrogen sources, a series of N-phenyl benzamides were obtained with the aid of cost-effective and readily available iron catalyst. Compared with the above work, this mild procedure featured room temperature, neutral condition, and simple catalytic system. During the conversion of benzylic alcohol, the corresponding benzaldehyde could be detected by TLC and GC-MS, indicating that the alcohol was oxidized to aldehyde in situ by nitrobenzene and then underwent the amidation. According to the detailed experiments, a tentative mechanism was proposed that iron species triggered the radical process and the solvent played a critical role as O-atom acceptor during the transformation.

**Scheme 48:**
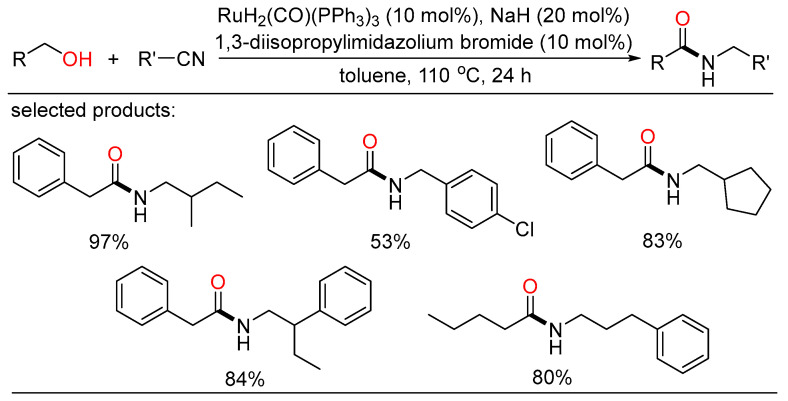
Ru-catalyzed amidation of alcohols with nitriles.

On the other hand, a completely atom-economical and redox-neutral catalytic strategy to amide synthesis between one alcohol and one nitrile was first established by Hong (Scheme 48) [109]. In the presence of NHC-based ruthenium catalyst, the amide C-N bond was constructed in one step without any byproduct formation. This concise reaction was an overall redox-neutral process involving hydrogen transfer from alcohol to nitrile, providing a characteristic way of amide synthesis with 100% atom economy.

**Scheme 49:**
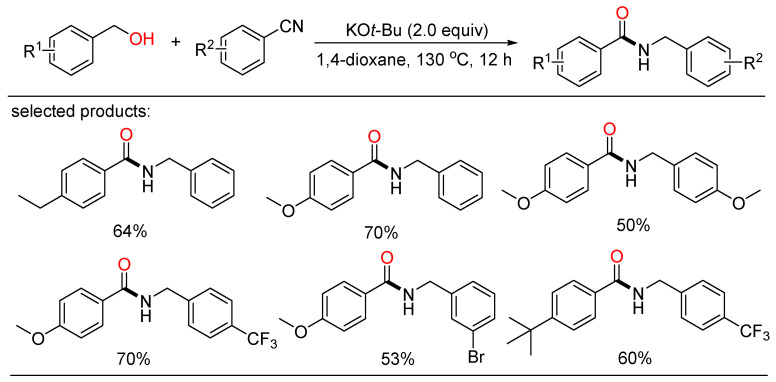
KO*t*-Bu-mediated amidation of alcohols with nitriles.

Lately, Fang and co-workers developed a simple protocol to amide formation enabled by Meerwein–Ponndorf–Verley (MPV)-type reduction from benzylic alcohols and benzonitriles under transition-metal-free conditions (Scheme 49) [110]. Using KO*t*-Bu as the base, a number of N-benzyl benzamides were readily gained with broad functional group tolerance. Mechanistic results implied that the transformation might undergo multiple MPV-type hydrogen transfer pathways.

## 5. Conclusions

As naturally abundant, low toxic, and cost-effective bulk chemicals, alcohols have been identified as good acyl surrogates instead of other common acylation reagents. Over the past years, tremendous progress in this field has been made to showcase an efficient, economical, and sustainable avenue for the construction of multiple acyl-containing molecules. This review discussed recent advances in using alcohols as ideal acyl sources to construct C-C and C-heteroatom bonds, thus yielding ketones, esters, amides, etc. Although this strategy shows remarkable highlights, many of reported protocols suffer from elevated temperature, stoichiometric external oxidants, and indispensable noble or complicated metal catalysts, which will likely preclude more widespread use in late stage applications. As a consequence, it is highly necessary to develop more prominent scenarios for these transformations under milder conditions. Fashionable synthetic styles such as photocatalysis, electrosynthesis, and mechanochemistry are anticipated to be further developed in this domain in the future.

## Data Availability

Not applicable.
